# The Complexity of Decreased Work Ability: Individuals’ Perceptions of Factors That Affect Returning to Work after Sickness Absence

**DOI:** 10.3390/ijerph19010113

**Published:** 2021-12-23

**Authors:** Ella Näsi, Mikko Perkiö, Lauri Kokkinen

**Affiliations:** 1 Research Unit, The Social Insurance Institution of Finland, Nordenskiöldinkatu 12, 00250 Helsinki, Finland; 2 Faculty of Social Sciences, Tampere University, Arvo Ylpön katu 34, 33014 Tampere, Finland; mikko.perkio@tuni.fi (M.P.); lauri.kokkinen@tuni.fi (L.K.)

**Keywords:** decreased work ability, returning to work, counselling, disability pension, partial work ability, rehabilitation, social benefits, musculoskeletal disorders, age discrimination

## Abstract

Much of what has been written about decreased work ability is based on quantitative studies and has been written from the perspective of professionals, service providers or authorities. In our qualitative study, we sought to understand how affected individuals themselves perceive and experience the multifaceted factors that are related to their decreased work ability. Sixteen individuals in Finland with musculoskeletal diseases (MSD) participated in semi-structured interviews. The participants were potential clients of a multi-professional service pilot model, the TOIKE Work Ability Centre. Narrative and thematic analyses were utilised. The study found that individuals with decreased work ability have differing perspectives towards returning to work and often complex life situations. Five distinctive groups were identified based on self-assessed health, work ability and orientation towards work or pension: (1) the Successful; (2) the Persevering; (3) the Forward-looking; (4) the Stuck; and (5) the Pension-oriented. Health problems, unemployment, age discrimination, financial difficulties and skill deficits were the major challenges of the interviewees. Furthermore, they perceived the service and benefit systems as complicated. The TOIKE service proved useful to some of them. However, many had not utilised it due to a lack of understanding of its purpose. Identifying the distinctive groups and their needs may improve interventions. Ultimately, this may help to achieve Target 8.5 of the UN Sustainable Development Goals, which advocates the right to employment for all ages and for those with disabilities.

## 1. Introduction

The ageing of populations leads to increasing proportions of old-age pensioners and higher morbidity among workers [1,2,3]. Therefore, many countries in the Global North are concerned about labour productivity and shortages, and worsening dependency ratios [3,4]. In this context, governments are interested in supporting sustainable working life [5] by preventing work disability [2,6,7] and increasing the retirement age [2]. Concurrently, Target 8.5 of the United Nations’ Sustainable Development Goals [8] addresses social sustainability, aiming to “achieve full and productive employment and decent work for all women and men.” It is of importance to this article that the target particularly notes those with disabilities.

In a perfectly sustainable working life, an individual with an injury or illness always recovers adequately and returns to work smoothly, but in the real world, not everyone can return to work in a timely manner [9,10]. A persistent health issue often is the main obstacle to returning to work or to finding a suitable new job [11,12,13]. Studies have found that health problems, particularly long-term sickness absences, are strong predictors of filing for disability pensions [2,14,15]. 

However, abundant research agrees that work disability and returning to work after sickness absence are complex phenomena that encompass many individual, work-related, service-related and environmental challenges [3,9,11,12,14,16,17,18]. The multifaceted nature of work ability and returning to work is covered under the World Health Organization’s biopsychosocial model of disability and functioning [19] and the multidimensional work ability model [7].

When returning to work is not possible, retiring with a disability pension is a way to make a clear early exit from the workforce. However, disability retirement is something that a person cannot resolve independently, as it can be issued only after intense scrutiny, after which it is determined that the individual has a medically confirmed illness, disease or injury that significantly restricts work ability [15]. Consequently, individuals with complex problems may be left “in between,” caught within a bureaucratic labyrinth of services waiting for decisions and making recurring claims for rehabilitation, benefits or a pension [12,14,20]. 

Due to its multifaceted nature, decreased work ability can be viewed as a wicked problem [12,21] because its causes can originate from many sources, its possible solutions are not clearly identifiable nor comprehensive, it keeps evolving and it requires cooperation across different governmental sectors [22]. Alleviating such a wicked problem calls for multi-sectoral and multi-professional collaboration. Concurrently, new kinds of multi-professional interventions and service models have been and are developed in Finland and abroad to alleviate partial work ability using a holistic approach [10,23,24]. This study’s underlying assumption is that although many people need individual, holistic support and counselling to return to work and to apply for rehabilitation and other services, suitable advisory services are either not always readily available, or they do not reach those who would benefit from them. Overall, the diversity of social security benefits and services offers support, but their complexity may confuse people and wind up hindering return-to-work efforts [14].

Researchers have expressed a need for more qualitative research to gain a deeper understanding of decreased work ability “from the ground up” [14], particularly individuals’ perspectives, thoughts and feelings [10,17,25]. According to Metteri [20], the value of individuals’ true-life perceptions is that they challenge and complement official information that authorities provide. 

Therefore, this qualitative study’s overall purpose was to enhance understanding about the phenomenon of decreased work ability by interviewing affected individuals, with an emphasis on how they perceive this multifaceted societal problem. The aim was to examine sick-listed individuals’ experiences regarding decreased work ability, their challenges in returning to work and the services and other factors that help or hinder them in their quest to resolve their situations. 

This study’s main finding was that individuals with decreased work ability have multifaceted problems, and that five distinctive groups could be identified based on the individuals’ self-assessed health and work ability, orientations towards work or pension and perceptions concerning the future. Health issues usually were the main hindrance to returning to work. Age discrimination and a lack of adequate skills or training also emerged as noteworthy challenges. Consequently, the interviewees’ needs in terms of support and advisory services varied, implying that services should be tailored carefully according to each group’s specific needs.

## 2. Materials and Methods

This study utilised a semi-structured phone interview method, followed by narrative and thematic analysis, to construct a holistic understanding of subjective human experiences and the meanings that people assign to their experiences with decreased work ability [26,27]. This study was explorative and theory-informed, as the theoretical framework was not determined explicitly a priori [28], but was based instead on a wider comprehension of earlier research on work ability and disability. The rather loosely structured theoretical framework allowed for an inductive, data-driven analysis of the interviews [29]. Määttä [25] previously took a similar approach. 

### 2.1. Study Participants

The interviewees comprised Finnish individuals with decreased work ability who had been receiving a sickness absence benefit for at least six months due to a musculoskeletal disorder (MSD). The 16 participants were recruited from a pool of 24 people whom a social insurance professional (at the Social Insurance Institution of Finland, Kela) advised to utilise a multi-professional pilot service model, called the TOIKE Work Ability Centre, during the first half of 2018. TOIKE aimed to enhance clients’ capabilities and opportunities for returning to work at Pirkanmaa and Southern Ostrobothnia hospital districts in Finland [30]. Their multi-disciplinary team included an occupational physician, rehabilitation counsellor, psychologist and social worker, who offered work ability assessments, counselling and guidance, and psychological services. However, only some interviewees contacted the TOIKE Centre. The reason for recruiting individuals from this pool was to acquire rich, qualitative data about decreased work ability and affected individuals’ service experiences and needs.

Special attention was paid to guarantee that the data were collected, stored and analysed in a way that keeps informants’ identities anonymous because the interviews focussed on sensitive issues, such as the participants’ health [27,31]. The Kela ethics committee evaluated and accepted the research plan in June 2018.

Potential interviewees were sent a letter in July 2018 that informed them about the research project and upcoming phone interviews. The recipients were told that participation was voluntary, their identities would not be revealed at any point and their answers would not be used for any other purpose than the current study [32]. Altogether, 16 individuals agreed to participate in phone interviews in August 2018. Each participant was asked specifically for his or her consent to be interviewed and to allow the interview to be audio-recorded for research purposes. Their consent was audio-recorded.

Four interviewees were women and the others men, but gender was not viewed as relevant in this study; therefore, the interviewees were given gender-neutral pseudonyms, such as Sam and Pat. However, their gender can be discerned through the use of the pronouns “he” and “she,” which were used for the sake of readability. Instead of exact ages, interviewees’ age ranges are reported. Four interviewees were in the “39 years or younger” age group, another four were in the “40–50 years” age group and the final eight were “51 years or older” (see Appendix A for descriptive information in Table A1. Interviewees’ profiles). Some of the participants lived in urban areas and some in rural areas, but the exact areas where they lived were not recorded. In Finland, both rural and urban areas have fairly equal access to social and health services.

Most interviewees had done physically demanding work [33] in agriculture, construction, logistics, manufacturing or maintenance. One had done physically demanding work in the hospitality sector, and two had done work that combined physical and mental demands in the health care and transport sectors. One interviewee worked mainly in secretarial work. Only four of the 16 interviewees had returned to their former jobs. One of the younger respondents had been accepted by a study program, where she started her studies for a physically less demanding vocation. Another respondent soon was going to start a work tryout. Most interviewees were receiving temporary financial benefits, such as sickness allowance, unemployment benefits, rehabilitation allowance, rehabilitation subsidy or partial disability pension. Appendix A (Table A1. Interviewees’ profiles.) describes each pseudonymised participant using eleven variables and individual summaries.

### 2.2. Qualitative Interviews

The average interview lasted about 25 minutes, ranging from 15 to 60 minutes. All 16 interviews were conducted in Finnish; only the quotations, which were used to illustrate the results, were translated into English during the reporting phase. 

The interviewer encouraged participants to express their honest opinions and original prevailing thoughts, allowing them to emphasise the points that they deemed important. The semi-structured interviews allowed the interviewer to ask open questions to acquire knowledge about the informants’ reasoning, opinions and attitudes. The interview guide included 29 questions, of which most were open-ended. The guide also included certain commonly used quantitative questions, such as the interviewee’s self-assessment of his or her health on a scale from 1 to 5. The interviewees also were asked to compare their current work ability with their best lifetime work ability with the work ability score, so that 0 represented full work disability and 10 indicated work ability at its best [34]. After each quantitative question, the interviewees were encouraged to explain and elaborate on their numerical answers. 

The interview guide included open-ended questions such as: What are the main reasons for you to be outside of working life? What is hindering returning to work? What would help you return to work? Other questions asked how the interviewee felt about the Kela phone call to inform him or her about the TOIKE Work Ability Centre, why individuals either did or did not contact the TOIKE centre and how they perceived the TOIKE service itself.

### 2.3. Narrative and Thematic Analysis of the Interviews

The interviewees’ answers were transcribed verbatim, but without details on intonation, pauses or stuttering. The focus was on content, rather than choice of words or tone of speech [26]. The data were transferred to the Atlas.ti program for analysis. 

The analysis primarily was based on inductive coding, used for narrative and thematic analysis, as explained below. However, the data first were organised roughly into a table laying out each interviewee’s background. A table of interviewees’ profiles (Appendix A, Table A1) was created with each interviewee’s age group, employment type and sector and main health concern(s). The table proved useful during the inductive qualitative analysis as a quick reference tool when comparing interviewees with each other. The table was refined further and expanded throughout the entire analytical process to include more specific information, such as the interviewees’ labour market situation, future plans, self-assessed health and work ability, success and readiness to return to work and whether they had contacted the TOIKE centre. After narrative and thematic analysis, a summarising column also was added at the end of the analytical process.

Using the Atlas.ti program, a data-based, inductive content analysis was conducted, i.e., the interviews were read carefully several times [29] and thereafter coded with the intention to code each comment without any limiting framework. However, the data collection and analysis processes were theory-informed, as the interview guide was based on previous literature and designed together with work-ability experts. During the open coding stage, “headings” or “labels” were assigned to each meaning unit [29,35]. Following Graneheim and Lundman’s [35] guidelines, single sentences usually were identified as meaning units, but sometimes codes were assigned to clusters of sentences or single words. The data were categorised, where appropriate, into positive (+) or negative (−), or onto a numerical scale. For example, the interviewees’ answers to whether they successfully had returned to work were coded as positive or negative responses. 

In the next step, compiling summarised narratives proved useful as an analytical device [32] to form a clear understanding of each interview and the similarities and differences between interviews. The ontological premise of narrative analysis is that an individual is an active subject who assigns meanings to his or her life and life events [26]. Narrative research aims to understand concrete events and individuals’ experiences of their world, actions and endeavours [31]. 

Therefore, each interview aimed to form a condensed narrative, including the interviewee’s vocational background and health issues, and their success in and perceptions of returning to work or orientation towards pension, re-education or rehabilitation. The summarised narratives also included their accounts of their main challenges and experiences with and perceptions of the TOIKE Work Ability Centre and other services. The narratives revealed more or less work-oriented career trajectories that allowed for organising the interviews into five distinctive groups based on the narrative analysis, serving as the core structure for reporting the results. For each group, one narrative was chosen to represent typical stories in the group. Relevant quotes illustrate the interviews’ original quiddities.

Concurrent with the narrative analysis, as the interviews were read many times, the initial coding was revised and specified. Codes were combined to form groups [29], termed code families in the Atlas.ti program. The codes and code groups then were organised into themes. The four most relevant emergent themes are reported at the end of the results section.

## 3. Results

**Figure 1:**
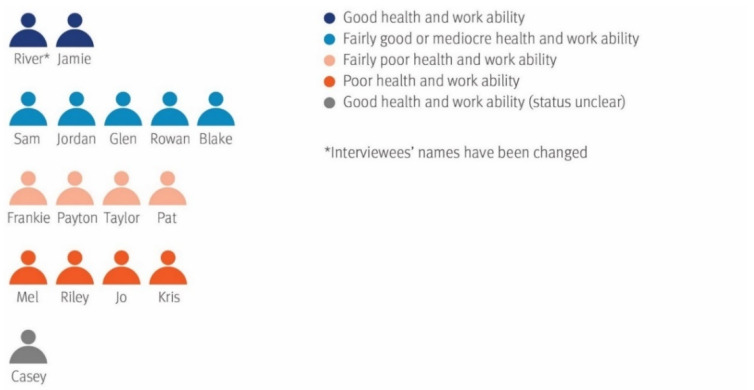
Interviewees’ perceived health and work ability.

**Figure 2:**
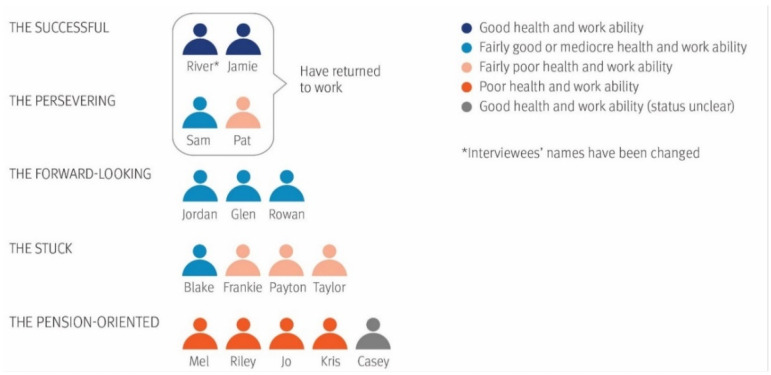
The interviewee groups based on their success in returning to work.

Figure 1 presents the interviewees’ self-assessments of their health and work ability, which serves as an introduction for reporting this study’s results. Thereafter, the study’s main findings are presented by explaining the five distinctive groups that were found to represent decreased work ability (Figure 2). These groups were categorised as a result of the narrative analysis process in which the interviews were rearranged into groups based on the interviewees’ orientations towards pension or working life, and their success in returning to work. Furthermore, four main themes emerged from the data in relation to supporting sustainable working lives.

### 3.1. Perceived Health and Work Ability Predicted Return to Work

The interviewees’ self-assessed health correlated rather well with self-assessed work ability. Only two interviewees felt that their health and work ability were good; they were assigned the pseudonyms River and Jamie and are depicted in dark blue in Figure 1. At the opposite end, four other interviewees—Mel, Riley, Jo and Kris—described their health as poor or very poor, and their work ability; they are depicted in orange. Two members in this group, Riley and Kris, itemised a long list of health problems or multiple body parts where they experience pain. Kris’ account illustrates the multimorbidity of an interviewee whose health and work ability are poor:

Kris: “*My back and right leg are duds. I have had sciatica. There is a problem in my pelvis, and my left bicep is loose. These are all occupational traumas. I am queuing for surgery, and they are saying: ‘Aren’t you going to work?’, even though the orthopaedists have written: ‘unfit for work’. Not in my right mind [in reference to not going back to work]! Additionally, then I have type 2 diabetes. Additionally, then I have had a hospital infection [nosocomial infection]. I cannot sit still, cannot sleep... I have medication for high blood pressure*.”

Those categorised as reporting mediocre or fairly good health and work ability are depicted in light blue in Figure 1, comprising Sam, Jordan, Glen, Rowan and Blake—also pseudonyms. Those reporting fairly poor health and work ability are depicted in light pink and include Frankie, Payton, Taylor and Pat. 

One interviewee, given the pseudonym Casey, had a health and work ability status that was difficult to categorise because she provided conflicting information. In the beginning, she assessed her health and work ability as good, but her account of her health is not logical. She claims that she was not ill, but recently went to a physician, complaining about health problems, and was receiving a sickness allowance. She is categorised in her own health and work ability group, called good health and work ability (status unclear) and is depicted in grey.

The narrative analyses revealed certain similarities and consistencies within the participants’ narratives, making it meaningful to categorise the individual narratives into five groups, as listed below and illustrated in Figure 2. Success in returning to work and orientation towards work or pension were the main factors in the narratives that shaped these categories. Furthermore, the interviewees’ open-ended answers regarding their health issues and recovery, and other factors affecting their motivation regarding returning to work, also impacted the grouping:

1. The *Successful*: work-oriented, returned to work, well-recovered and -motivated: River and Jamie
2. The *Persevering*: somewhat work-oriented, returned to work, not fully recovered, concerned about their work ability: Sam and Pat
3. The *Forward-looking*: work-oriented, have not returned to work, not fully recovered, motivated to find a new job: Jordan, Glen and Rowan
4. The *Stuck*: somewhat pension-oriented, have not returned to work, not recovered, passive: Blake, Frankie, Payton and Taylor
5. The *Pension-oriented*: have not returned to work, not recovered, demotivated: Mel, Riley, Jo, Kris and Casey

In fact, self-assessed health and work ability can be viewed as one of the primary factors affecting an individual’s categorisation into a specific group, as demonstrated in Figure 2. Thus, interviewees’ better self-assessed health and work ability were associated with a stronger orientation towards and better success in returning to work. Nevertheless, it is worth noting that the correlations were not complete due to the complex nature of decreased work ability. Interviewees who returned to work (the *Successful* and *Persevering* groups) or who were hoping to return to work (the *Forward-looking* group) mostly assessed their health and work ability on the positive side. Accordingly, those who were applying for a disability pension and had difficulties finding a job that would suit their health constraints (the *Pension-oriented* and *Stuck* groups) assessed their health and work ability in most cases as being poor or fairly poor. 

However, there were two exceptions to the aforementioned rule. Blake belonged to the Stuck group even though he assessed his work ability as mediocre. He fell and hurt his shoulder, and mentioned having “the blues” occasionally, but otherwise he did not have major health issues. Blake’s stagnant situation also was associated with his work history in unstable jobs. He explained that one solution could be re-education, but that his inability to use computers made it difficult for him to access relevant information, and he also was sceptical about the usefulness of studying. In relation to this, he alluded to low self-confidence in his interview answers. 

Pat’s narrative was a different type of exception to the rule that good health is a precondition for returning to work. He explained that he felt it was necessary to return to work and was also willing to go back, despite his fairly poor health and work ability. Pat’s use of the verb “must” when talking about returning to work indicates some level of pressure to return to work, which could be related to him being self-employed, even though he did not use entrepreneurship as an explicit explanation for his prompt return to work. Next, we analyse one by one all main narratives presented in Figure 2.

#### 3.1.1. The Successful Group: Well-Recovered and Returned to Work

Jamie, an entrepreneur, and River, a professional in the health care sector, were placed in the *Successful* group. They recovered well from their illnesses and returned to their old jobs. They were relatively free of pain as well. Jamie’s story was chosen to illustrate this group. 

Jamie worked in the agricultural sector, in which workloads are seasonally cyclical. He injured his shoulder at work, underwent surgery two months later, then returned to work four months after that. He explained that he himself carried the main responsibility for rehabilitation, actively exercising the injured shoulder under the care of a physiotherapist. Seven months after his operation, Jamie was happy with his recovery and enjoyed work: “*Then it [the shoulder] was operated on, and it feels reasonably good now. It is possible to work [...] It doesn’t impede my work tasks in any way.*”

When asked about his health, Jamie explained that it was rather good for a man his age, about to turn 60. He assessed his work ability as 8 on a scale of 0–10 and justified it through improved skills: “*My skills have increased, even if my pace has slowed down a bit. Skills have improved in any case.*”

One factor that may explain Jamie’s eagerness to return to work is that as a self-employed worker in the agricultural sector, he had firm control over his responsibilities, with success or failure having direct consequences on his financial rewards or setbacks. Furthermore, he also pressured the health care professionals to operate on his shoulder promptly to ensure fast recovery. This implied that he was determined to return to work and was motivated and committed to taking care of his work responsibilities: “*I said there is a need to get into surgery as soon as possible because I work in agriculture and the approaching sowing time is putting pressure on me. Additionally, I got [into surgery] very fast then.*” 

Jamie’s quotes above indicate that he felt a desire to return to work, which he found meaningful and important. Both individuals in this group recovered well due to comprehensive medical interventions and personal engagement in their rehabilitation. They did not feel the need to contact the TOIKE Work Ability Centre and were determined to return to their old jobs. Therefore, the following adjectives describe this group: work-oriented; well-recovered; and motivated.

#### 3.1.2. The Persevering: Returned to Work despite Work Ability Concerns

The *Persevering* group included two individuals who returned to work regardless of their prevailing health problems. Sam and Pat both were engaged in physical work in the construction sector. Sam was an employee, and Pat was self-employed; both experienced back pain. Sam added that he was suffering from burnout/fatigue. 

Sam’s story is provided here as an example to illustrate the situation when an individual returns to work despite not having recovered from a disease or disorder. Sam was in the 40–50 age group. He presumed that his physical and mental health problems, back pain and burnout/fatigue may have been interconnected. To cope, he turned to an occupational health care service for his back problems and a psychiatrist for his mental health problems. He was on medication, did physical exercises and felt a need to rest. Sam also sought and got help from traditional medicine and acupuncture. Despite multiple periods of sick leave, Sam returned to work, which can be interpreted as a sign of being work-oriented. He also assessed his health as mediocre and his work ability as 5.5 on a scale of 0–10, i.e., despite the pain, he felt that he still possessed work capabilities. However, he did not have much choice and said that he had not received much information about rehabilitation: “*The choices that have been available are: ‘Be on sick leave or go to work’. ‘Take some medicine if you have pain’. This TOIKE is the only thing I’ve received info about.*”

Sam contacted the TOIKE Work Ability Centre. He was confused after not being offered an appointment with them and was told that due to having access to occupational health care services, there would have been little extra benefits available for him from the TOIKE Centre. However, he had some unresolved work-ability issues, so it is likely that he could have benefitted from a holistic advisory service to ensure that his work ability issues were eased, rather than worsened or prolonged. 

Both Pat and Sam had many years of potential working life ahead of them, as they were in their 30s and 40s, but they had prolonged health problems that posed a risk of lost work ability in the future. They both worked in the construction sector, which is physically demanding. To sum up, this group of work-oriented individuals can be described as: returned to work; not fully recovered; and having prevailing work-ability concerns.

#### 3.1.3. The Forward-Looking: Searching for New Opportunities

The interviewees in this *Forward-looking* group were seeking a new direction for their careers. They all had musculoskeletal disorders, which inhibited them from returning to their old jobs, but conveyed a strong orientation towards finding suitable work. They include Jordan, who had worked in the hospitality sector; Rowan, who had worked in the transport sector; and Glen, who had worked in logistics. All three were under age 40. They expressed hopefulness, anticipation of recovery and a willingness to find new career paths that would fit their health constraints. 

Glen’s story illustrates this group. He had been working in a physical job, and his shoulder and clavicle were injured in a violent incident. The injury was rather serious and required two shoulder operations, but the operations were not very successful, and his absence from work due to incapacity lasted a year. His recovery advanced slowly, and he found physiotherapy helpful, but could not return to his old job. He assessed his life satisfaction as mediocre now and envisioned finding a new job or seeking training or education to increase his satisfaction: “*I cannot really do physical work, even though I liked it very much. When I get a job or school [...] or something else, then that will, of course, affect [my life satisfaction] very much in general. I miss working life in a way, going to work and so forth. Even though it is hard, but still.*”

Glen recently turned to a nongovernmental organisation (NGO) that offers work tryouts and other types of activities to unemployed people. He hoped to find education or work soon: “*I have tried to make future plans by myself. I have been thinking that I will apply to some college or training in the autumn and try to find a job also at the same time. It is possible that the TOIKE thing would still be useful, but I have not contacted them yet.*”

None of the members of this *Forward-looking* group expressed a preference to remain outside of working life. They all were looking forward to finding new directions for their careers, but none had contacted the TOIKE Work Ability Centre. They all had prevailing musculoskeletal disorders, but viewed recovery and regaining work ability as plausible and desirable future trajectories. Thus, this group can be referred to as work-oriented—not fully recovered and not returning to work yet, but looking actively for a new direction.

#### 3.1.4. The Stuck: Having No Clear Exit from Temporary Benefits

The four interviewees in the *Stuck* group—Blake, Frankie, Payton and Taylor—had injury, pain or disease of the upper extremities, and some also had other health problems as well. They had worked in physical jobs to which they had not been able to return. However, they mostly assessed their health and work ability as mediocre or somewhat diminished, rather than highly diminished, and they felt they had some capabilities left. Two were in the 40–50 age group, and two were in the 51+ age group. Taylor and Frankie had been absent from work for six years, Blake for two years and Payton for half a year.

Taylor’s story was chosen to represent this group. Taylor lived in a small town, had worked in manufacturing and was unemployed with a shoulder injury. He stated that his decreased work ability was a hindrance to finding new employment: “*I have been trying to look for such a job in which I could work with my arms down. I cannot perform tasks where I need to reach upwards with my arms—that is the kind of work I have always done. My arm just does not work upwards.*”

Before injuring his shoulder, he had been unemployed for many years already. When asked about returning to work, he mentioned many factors besides his shoulder injury that have hindered his job search efforts, such as learning difficulties, deterioration of his general health, lack of education or training and ageing. He also cited his place of residence and living conditions as factors that made it more difficult, or even impossible, to find suitable work.

Taylor: Studying doesn’t help because I have dyslexia and memory disorders, so that things go in through one ear and out the other. My health condition in general is getting worse. I don’t have training for almost anything, and the other thing is that my shoulder is a wreck. These are the two most important reasons. Additionally, then this house—I really cannot go far [...] You know that I am an old bloke already. I do not have such dreams anymore as I had when I was in my 20s.”

Taylor had multiple health, social and location-related problems. When asked what he thought about the possibility of contacting the TOIKE Work Ability Centre, he ignored the question at first: “*It is possible that somebody called me [referring to a Kela professional calling and recommending TOIKE]. Sometimes I feel that so many magazine peddlers call me, so I don’t care about them [...] Oh, that thing in Tampere [refers to TOIKE]. I did not contact them.*”

A common feature of this group was that these individuals did not express a clear preference for retirement, nor much hope for returning to work. For this group, these individuals’ prominent health problems were not the only reason for decreased work ability, as they reported a complex mesh of hindrances. Making a living was a challenge for this group. Three out of four cited lack of job opportunities as a major reason for not returning to work.

Another common feature in this group was their unwillingness or inability to acquire new skills. Similar to Taylor, Frankie also claimed that he did not have an adequate, up-to-date education. Frankie also revealed that he had dyslexia in addition to his physical health problems. 

Additionally, the following problems came up during the interviews: Their subsistence relied on temporary benefits, they experienced difficulties navigating the benefit and service system, and their financial situations were poor, i.e., they were stuck in a rut of temporary benefits that they could not exit to working life or a disability pension. Overall, a certain level of ineptitude in seeking resolutions to their work disability status was present during these interviews. To summarise, this group comprised people who were somewhat pension-oriented, had not returned to work, had not recovered and were passive and hesitant.

#### 3.1.5. The Pension-Oriented: Inclination towards Early Retirement

Four interviewees given the pseudonyms Mel, Riley, Jo and Kris, who had poor health and work ability, and Casey, who provided contradictory information about her health, comprised the *Pension-oriented* group. 

Mel, Jo, Kris and Riley were all over 50 years old, had done physical work and had chronic musculoskeletal problems that caused pain and decreased their functioning. None of them had returned to work and expressed a clear reluctance towards returning, as they viewed themselves as unfit. Jo, Kris and Riley had applied for a disability pension. Mel did not mention any pension application, but did not believe that he could—nor did he want to—return to work. Their main reason for being outside of working life was their poor health. They all mentioned having surgery as an option to recover, but had differing perceptions of surgery’s appropriateness for themselves. Kris, Riley and Mel were scared or reluctant to take the surgical route back to work. However, Jo welcomed the prospect of surgery and viewed it as the only solution to his work-ability issues, but as he had assessed his readiness to return to work as zero (on a scale of 0–10) and had applied for a disability pension, it is plausible to categorise him as pension-oriented. 

The manifest content of their interviews, including thorough accounts of persisting health problems, affirmed that they were incapable of work; therefore, a disability pension could have been a plausible option for them. It may be worth nothing that a risk of justification bias [36] is present in studies that ask participants to self-report their health: Inactive individuals may report poorer health to justify their situation. Kris and Riley aimed to justify staying outside of working life after having worked very hard in the past:

Riley: “Well, I would be happy to return to work if I was healthy. I have always liked to work, but maybe I worked a bit too much because I am a wreck. We pushed night and day then. We did not have any summer holiday or anything. Heavy work. Twelve hours was our shortest day. Then my body started to break up.”

Kris’ story was chosen as an example to illustrate this group. Kris had worked as an entrepreneur in the construction industry. He ended his business three years earlier due to back and leg pain, and diminishing business opportunities. He also had multiple morbidities and received medical statements from orthopaedists about his decreased work ability. His own assessment of his work ability was zero, and he was applying for a disability pension. However, Kris’s pension application was unresolved for the time being, as he was scheduled for surgery in the near future: “*I am a bit [...] multi-handicapped. I should have gone 10–20 years ago to get treated, so maybe something could have been cured. I let myself [get in a bad condition], was stupid and worked too much.*”

Kris explained that he did not go to see a doctor immediately after ending his business and implied that this delay was the reason why he had been denied certain benefits that he was expecting to receive from the pension insurance company. Kris relied on basic social assistance to make ends meet. He complained about delays in getting sickness allowance payments, which had aggravated his poor financial situation. He felt frustrated and angry due to these difficulties in accessing social benefits and regretted working too hard, not taking care of his health and not seeking medical care early enough. He felt betrayed and blamed the bureaucracy for his difficult financial situation: “*The last time I received any money was more than a month ago. I am so angry. I am saying I could sue them. I need to visit the hospital often, but I cannot even pay for it [the hospital bills].*”

Casey was a somewhat different case because her health status was hard to determine due to conflicting information. She was over 60 years old, had worked in various secretarial jobs, considered herself fit for work, but could not find work. She explained that she was advised to apply for a sickness allowance in relation to pain that she had experienced and stayed home for many years caring for her husband until he died*:* “*I had a stomachache, and I pointed out that it stems from here, between my ears. I pointed at my head, and the doctor asked: ‘When did the headache start?’ Additionally, she made a diagnosis that I have a problem in my head. The problem was in my stomach. So, I have come across these bafflements that I was prescribed medicine for an affective disorder. I did not fetch the pills because it was my stomach that was aching. [...] There is nothing wrong with my health. For two years, I cared for my husband, who had a brain tumour. They just labelled me ill.*”

It was difficult to draw reliable and consistent conclusions from Casey’s interview transcript. She may have either had a stomach problem or an affective disorder, or both, or her unclear account could have been related to some other physical, mental, cognitive or memory disorder. It is also possible that she intentionally or unintentionally used confusing language at the doctor’s appointment and during the interview as a consequence of disappointments or frustration in relation to her difficult life situation.

Casey lived in a small town and mentioned diminishing job opportunities as one reason for not returning to work. However, the employment office required her to participate in activation measures, so they sent her to jobs. For example, she had worked in the city library, where she recalled “*going through books in the storage of the library’*. She felt that the jobs she was assigned were “*nonsense*” because she was given tasks that required fewer skills than what she was capable of doing. 

Casey recalled a Kela professional contacting her and stating that she will be “*put into rehabilitation’*. In response, Casey attempted to legitimise her inactivity in the labour market as making space for younger workers: “*There could be an age limit because, you see, I don’t see it as useful to try to rehabilitate over-60-year-olds back to work. I am not so young, so is it sensible to employ me? I think, in this situation, it would be more reasonable to hire, for example, someone who is 34 years old.*” In accordance with her disdain for rehabilitation, she had not contacted the TOIKE Work Ability Centre. 

To summarise, this last group can be called the *Pension-oriented* group because these individuals had not returned to work and were expressing demotivation toward rehabilitation or activation measures and having to return to work.

### 3.2. Supporting Sustainable Work Ability

In addition to identifying the aforementioned five different groups among the individuals with decreased work ability through narrative analysis (see Figure 2), the following topical themes on supporting sustainable work ability also emerged from the interviews through thematic analysis.

#### 3.2.1. Age and Skills as Factors Affecting Returning to Work

Some interviewees mentioned their age as being a factor even though they were not specifically asked whether they think their age affects their work ability or job opportunities. For example, Blake mentioned age while explaining his difficulties coping with strenuous work and his low motivation to train for a new vocation: “*My work has always been strenuous, and now I cannot move or lift anything with my left arm. I should have this arm treated or then I should change into a different vocation. However, I don’t know. I am aged already.*”

Casey’s main explanation for being outside of working life was age discrimination in the labour market: “When I became unemployed, I was already too old for the labour market, let alone today. I will be 61 next week. I can tell you that there is nobody in this town who would hire me.”

In addition to Casey, almost all the other interviewees who were over age 50 had given up their hopes and dreams of returning to work. These older individuals had in common that they felt their health and work ability were poor. 

Finding work that could be adjusted to accommodate their lighter requirements was difficult, particularly among those over 50 years old. In fact, for the *Stuck* group, difficulties in finding suitable work were equally or an even more serious problem than the health problem itself. Jamie was the only exception among the interviewees over age 50, as he was very motivated to return to work. His case was viewed as an example of *the Successful* group, and his motivation was explained by his well-advanced recovery and meaningful entrepreneurial work. 

Having outdated or insufficient training or skills also can be factors that hinder returning to work. For example, Taylor, from the *Stuck* group, attributed his joblessness to a lack of skills, among other things: ‘*I don’t have training for almost anything’*. Another interviewee who had given up hope was Frankie, who had been outside of working life for six years: “*I do not have any vocation at the moment. I have been unemployed for a long time. Actually, I have not been unemployed, but outside of working life.*”

On the contrary, those in the *Future-oriented* group, who were all under age 40, were hopeful and eager to find new directions for their careers and motivated to go through re-education if necessary.

#### 3.2.2. The Importance of a Coherent Health Care Service System

The interviewees who reported having easy access to occupational or specialised health care services and who were content with the care and advice that they had received also returned to work. For example, River, who had severe back pain, recovered and returned to her old job due to having a solid doctor-patient relationship. She also was motivated to participate actively in physiotherapy and searched for information about her condition in the literature. Perhaps her background in health care contributed to her ability to search for and comprehend relevant information and commit to the suggested exercise routines. When asked about her own efforts at recovery, River explained: “*I have a very good and close relationship with my doctor. It gave me a lot. Additionally, then I also go to see a private specialist—to get a second opinion. Recovery started and the likelihood of going back to work became clear to me. That I can return, and I will recover. I felt that I don’t need anything like that [refers to the TOIKE service]. Active physiotherapy is enough.*”

By comparison, Rowan, who had been looking for a new direction independently, recalled incoherence in accessing services. He was a young adult in need of holistic health care services, as he mentioned having “other problems” in addition to his main diagnosis, which was back pain. Rowan’s quote illustrates the situation of being “bounced” from one authority or service provider to another: “*I would need a health care service provider who would examine my health as a whole and not consider only one thing at a time. I find it very troublesome because you need to go to different places. With every health issue, you need to have a separate appointment.*” 

Furthermore, being self-employed and, thus, personally responsible for acquiring occupational health care services may create situations in which potential risks can be anticipated. For example, Pat did not utilise the services of a physiotherapist, but instead tried to manage without, even though he was an entrepreneur doing demanding physical work and suffering from prolonged back pain: “*Perhaps I should think about ergonomics.... I have bought tools that could help.*”

#### 3.2.3. The Challenges and Potential of Multi-Professional, Holistic Counselling

In addition to health care services, the interviews indicated that individuals with decreased work ability often needed support from other service sectors, such as social security, rehabilitation and employment services. For example, Rowan also could have benefited from career counselling and vocational rehabilitation because he felt that he was unable to return to his old job due to back pain. These issues were all included in the service package that the TOIKE Work Ability Centre provided. A Kela professional had advised Rowan to contact the TOIKE centre, and he thought that its multidisciplinary counselling could be the kind of holistic service concept that could help him. However, the inconsistency of service paths is disclosed in Rowan’s incident: “*In hindsight, I feel that the TOIKE project would have probably helped me. It is probably a service that I would have contacted if these other things had not been around. Maybe the most decisive thing was that there was someone in the health care services who specifically told me that it may not be the right time now, but when things have settled a bit, I should contact them [TOIKE].*”

In addition to Rowan, also other interviewees found the service systems to be fragmented and bureaucratic. The interviewees were not always aware of what kinds of services were available, which kinds they were entitled to and how to apply for them. Sam’s quote illustrates the obscurity: “*One expert advised me to do something and then another said the opposite [...] They ask me to provide all kinds of things [forms, applications]. Additionally, then I don’t even know what I am supposed to send them, and I send them things without knowing what I am sending. Very unclear. Extremely unclear.*” 

The interviews indicated that information about new multi-professional services’ purpose is not always clear to potential clients, which may lead to avoidance of services designed to help and support clients. For example, Kris and Riley did not want to contact the TOIKE Centre because they anticipated that they would be pressured to go to work.

Riley: “I am on sick leave now. Immediately, they try to activate me. Otherwise, it would be OK, but pain takes away all my strength. I wouldn’t mind [contacting the TOIKE], but they immediately started to bombard me with all kinds of requirements. Luckily, I was offered the right to use the gym here. I am content with that. My functionality is low, and the constant pain gets on my nerves.”

Payton and River also got the wrong impression from the TOIKE Work Ability Centre. They thought that the primary aim was to guide them toward new vocations, which they wanted to avoid, because they were committed to their current vocations. River’s reaction to the Kela phone call, during which she was told she could benefit from career counselling at the TOIKE Work Ability Centre, illustrates the clash between what she thought was offered and what she felt she needed: “*First, I felt confused because I have a permanent job, and I hope they are not trying to direct me somewhere else.*”

Despite accessibility challenges, many interviewees mentioned the importance of personal advice that professionals provided. Some received expert guidance and advice from their doctor, occupational health nurse, Kela or the TOIKE Work Ability Centre. For example, Rowan, whose discouragement regarding contacting TOIKE was explained earlier, had received useful advice from a Kela professional. He explained that the Kela phone call gave him a lot of information on vocational rehabilitation and benefits. He was expecting to start a job tryout soon.

Mel and Frankie had visited the TOIKE Work Ability Centre and were satisfied with the multi-professional service there. Mel, who had been outside of working life for six years, said he received good advice from TOIKE. He was advised on how to apply for a partial disability pension and felt that it was best to comply with the advice from professionals to continue receiving benefits: “*I have visited there [TOIKE] a couple of times. I hope Kela is satisfied now that I am doing what they want. I have been in a job tryout through the pension insurance company, but it did not turn out well. They just want to boost their own egos by trying to organise work for me, but they do not consider my situation…. I am not fit for work.*”

#### 3.2.4. Exits: Justified Career Orientations

**Figure 3:**
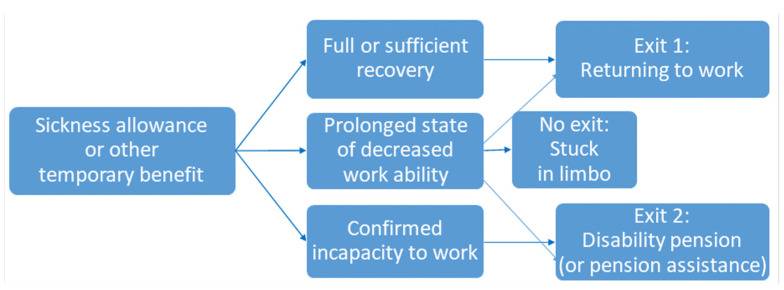
The exit path model. The primary exit from temporary benefits is returning to work. The secondary exit is retiring with a disability pension. (Besides, persons who are over 60 years and unemployed for five years with no or few interruptions, may be eligible for pension assistance, equal in amount to the minimum quarantee pension, in Finland.) If these exits are not available, the individual may be stuck in limbo, circulating between different temporary benefits.

The interviews highlighted two desirable “*exit paths*” for individuals from their unresolved life situations (Figure 3). First, for the well-recovered individuals, it is possible to return to work (Exit 1). Second, accessing a disability pension may enhance well-being significantly, particularly among those with the widest disability deficits and advanced age (Exit 2). The analysis demonstrated that the fate of a group with prolonged partial work ability is often—at least temporarily—no exit. These people are left to circulate between service providers and welfare schemes, and may end up applying for and relying on recurring temporary social security benefits, such as unemployment benefits, sickness allowances, rehabilitation allowances and non-permanent disability pensions.

In this study, those in the *Successful* and *Persevering* groups found their way back to work, i.e., Exit 1. However, those in the three other groups were currently in situations with no clear exit yet. Those in the *Pension-oriented* group clearly were yearning for retirement, i.e., Exit 2, and those in the *Future-oriented* group clearly were looking for a new career, i.e., Exit 1. Those in the *Stuck* group were not expressing a clear preference toward either exit, but instead described their personal situations as being awash in confusion and a lack of clarity.

## 4. Discussion

This qualitative study’s results clearly demonstrate that individuals with decreased work ability are a heterogeneous group with complex problems. However, it is possible to identify sub-groups within them. This corresponds with earlier research, e.g., Heikkinen’s [37] register-based research on similar study participants in Finland and Lydell et al.’s [17] qualitative study in Sweden. The interviewees in this study expressed many different factors, besides health, that affect returning to work. They revealed individual factors such as advanced age and insufficient skills, work-related factors such as temporary contracts and demanding work tasks, service-related factors such as delayed social benefits or fragmented health care services and environmental and social factors such as remote living areas and family responsibilities.

### 4.1. Main Findings

This study’s main finding is that even though individuals with decreased work ability are a heterogenous group, it is possible to categorise them into distinctive groups based on their orientation and success in returning to work. This study’s interviewees provided a collection of unique narratives that included certain similarities in the factors affecting their future career prospects. A comprehensive, data-driven, narrative analysis of the interviews identified five groups among the individuals whose work ability was decreased due to musculoskeletal disorders: (1) the *Successful;* (2) the *Persevering;* (3) the *Forward-looking;* (4) the *Stuck;* and (5) the *Pension-oriented*. 

Another clear finding from previous studies and this study alike is that the individual’s self-assessed health and work ability is a useful indicator for predicting success in returning to work [38]. Health problems tend to shift individuals towards early retirement, but in addition to health and work ability problems, other individual, work-related, social and environmental circumstances were contributing factors in study participants’ success in returning to work.

### 4.2. Limitations

Using data from 16 interviews to elicit data-driven narrative analysis, this study identified five distinctive groups among individuals with musculoskeletal disorders based on their self-assessed health and work ability, orientations towards work or pension and perceptions concerning the future. All of the potential interviewees had been advised to contact the TOIKE Work Ability Centre due to an MSD and decreased work ability. However, there might have been some differences between those 16 who agreed and those eight who did not agree to participate in the study. Due to lack of data on non-participants we had no means for evaluating whether they had some common characteristics different from the participants, which could have possibly led to finding more categories than we did in the current study. In future studies, these findings should be tested with other conditions, such as mental health disorders, and with larger data sets from different countries to see whether the typology is generalisable to different study designs and populations. 

### 4.3. Interpretation and Implications

The interviews reaffirmed findings from previous literature that advanced age is a factor in increasing pension orientation and obstacles related to returning to work, particularly among individuals with prolonged sickness absences [17,25]. Older people also face difficulties in finding work due to age discrimination within the labour market [2]. Ageing is associated with decreased work ability and increased morbidities [1]; therefore, work can become physically or mentally burdensome at an older age. 

The pension orientation conveyed in the interviews usually was derived from negative experiences and delays in rehabilitation or health care, being rejected or discriminated against because of advancing age, or enduring persistent physical pain and feelings of decreased self-efficacy [11]. Furthermore, an interviewee with a stagnant life situation was likely to be associated with a work history comprising unstable jobs [13], and his scepticism about the usefulness of re-education corresponds with Jahoda’s [39] argument that the self-confidence of people outside of the workforce often decreases because they feel ashamed and start to question their skills and capabilities [17]. Conversely, those who belonged to the *Successful* group and returned to work described how they had a meaningful job, actively had been seeking medical help and were dedicated to conducting all the exercises recommended to them as rehabilitative measures to support a prompt recovery. This supports the assumption that timely rehabilitation [17,40] should be more readily accessible to all individuals with decreased work ability to stop a possible negative trend of deteriorating health and work ability.

A closer look at the situations of those in the *Pension-oriented* group revealed that their situations were complex: They had multiple morbidities and contextual hindrances, such as outdated professional skills, a history of unstable work contracts, having gone bankrupt or living in a small town where job opportunities were scarce (see [13], for similar circumstances). Additionally, due to their advanced age, they were in the most disadvantaged situation in the current labour market, in which competition for job vacancies is harsh, and age discrimination exists [2]. Consequently, they had given up hope of returning to work and instead clearly were inclined towards retirement. For them, a justified and timely exit with a disability pension could be the difference between a disappointed “in between” life and one of well-being within the limits of their remaining functionality.

The interview data brought up the relevance of labour market dynamics on individuals’ career trajectories. Many within the *Stuck* and *Pension-oriented* groups explained that there was a clear lack of job opportunities that accommodated their decreased capabilities, with fewer jobs available in the areas where they lived. Furthermore, they communicated that their skills and education were outdated regarding the current demands of working life. Conversely, interviewees in the *Successful* and *Persevering* groups explained how a desire to work pulled them back into their jobs, which they deemed meaningful, important or necessary. 

Therefore, this empirical study’s findings are in line with the biopsychosocial approach of the International Classification of Functioning, Disability and Health [19] and the multidimensional work ability model [7]. These holistic models contend that work ability depends not only on the individual’s health, but also on their functionality, education and skills, work demands and environment, family and community support, and governmental support and services. This study reinforced the idea that labour market dynamics influence how strongly work pulls the individual back into the workforce or whether current economic situations and stringent competency requirements push employees with decreased work ability or outdated skills out of the workforce [38,41,42]. 

This study’s empirical results, together with previous literature [43], imply that it is important to emphasise enhancing individuals’ capabilities through lifelong learning. A requirement for sustainable careers in today´s working life is that everyone’s skills are updated regularly. Of course, this would require substantial input from society, employers and individuals themselves.

As this study adds to the existing literature on decreased work ability’s complex nature, it is plausible to assert that decreased work ability is a wicked problem. Decreased work ability’s wickedness can be elaborated through the concept of the triple burden of decreased work ability, i.e., the complex mesh of problems present in the *Stuck* and *Pension-oriented* narratives in this study. The three main burdens suppressing the partially disabled are: (1) health problems; (2) lack of job opportunities; and (3) financial distress. When these burdens are combined, they create a vicious cycle that is difficult to break (see [25]), in which the individual is not empowered to utilise the capabilities (see [44]) that he or she has left. 

The theoretical contribution of this thesis is *the exit path model* based on previous literature [12] and this study’s empirical results. The exit path model simplifies the alternative working-life prospects of individuals with decreased work ability into two clear exits—(1) *returning to work* or (2) *retiring with a disability pension*—and an additional unclear option of remaining in limbo between resolutions and having (3) *no exit.* Additionally, other studies [17,25,37] identified this kind of stalemate situations among the participants in their studies. The primary exit from temporary benefits is returning to work, as working supports health and well-being. The secondary exit is retiring with a disability pension, which ends uncertainty and eases the person’s stress caused by feelings of enduring a temporary status and related social benefits. However, if a clear exit is not available, the individual may be locked into remaining in limbo, shifting to and from various temporary benefits [12,14,37]. 

This exit path model proved useful in presenting the different situations and service needs of the five groups found among this study’s interviewees. Those in the *Successful* and *Persevering* groups utilised the primary exit and returned to work, but it is likely that those in the *Persevering* group, who returned to work with perpetual work ability concerns, could have benefited from rehabilitative services such as physiotherapy, assistive technologies or psychological methods of pain management. These would be important in ensuring that their work ability improves, rather than declines, in the future. Those in the *Forward-looking* group currently were on temporary benefits, but their focus was on returning to work. They expressed hopefulness and motivation, implying that for them, the preferred exit—finding suitable work—seems plausible. It may be justified to assume that for those in the *Forward-looking* group, perhaps the most essential service would be career counselling to pave their paths towards new careers. 

Meanwhile, those in the *Stuck* and *Pension-oriented* groups currently seemed to be stuck “in between” without a clear exit that would have resolved their situations. Some interviewees in the *Stuck* group still could have had a chance to navigate their way back to work. However, as their situations were complex, they would have needed appropriate rehabilitation and new work opportunities in which work demands would have been adjusted to their decreased capabilities (see also [25,45]). They would have also benefited from multi-professional advice in many aspects, such as re-education, health care, pain management and applying for services and benefits. 

By comparison, based on the perceptions and narratives of those in the *Pension-oriented* group, it may be justifiable to conclude that their well-being would have been served best by allowing them to utilise the secondary exit of retiring with a disability pension. They conveyed many factors that contribute to substantially decreased work ability: multimorbidity; advanced age; outdated competence; and a lack of job opportunities. 

In ideal situations, health care and other services support the returning-to-work process, but some individuals instead felt that they were turned down or passed from one authority or service to another (see also [25]). Health care services in particular were experienced as a fragmented system in which the individual felt like they were being “bounced” from one service provider to another, with overly long waiting times. The TOIKE Work Ability Centre plan viewed this problem as an important issue that needs to be tackled [30].

Furthermore, Määttä’s [25] qualitative data analysis of citizens’ accounts of their experiences with defects in the social security system uncovered similar situations of delayed access to services or denied benefits and consequent feelings of betrayal and grief. Kris’ narrative also could be categorised as an example of an “unfair and unbearable situation” that Metteri [20] uncovered in her analysis of situations in which the promise of the welfare state was not fulfilled for individuals who fell through the security net.

The hesitation and misconceptions that some interviewees had towards the multi-professional TOIKE centre imply that these potential clients had not assimilated the information on the forms of advice available. Thus, it is important that communication and promotion of advisory interventions be adjusted to clients’ individual needs. Some clients could benefit from career counselling or re-education, others could benefit more from advice on ergonomics and assistive tools, and some could be better served by helping them apply for a disability pension, rehabilitation or other benefits and services. All these forms of advice would have been available at the TOIKE Work Ability Centre, but not all potential clients were aware of them. These challenges regarding information and communication also have been reported in previous studies. For example, Aalto et al. [46] have suggested that information on available services should be increased in conjunction with the development of integrated care alternatives. Additionally, the findings from a study conducted in the UK [18] stressed the importance of role clarity, particularly clients’ needs for sufficient information about the services that they are offered. Therefore, in accordance with acknowledging differen tiated service needs and offering individually tailored interventions [17], service providers also should invest in approaching rehabilitation prospects with tailored messages. 

Additionally, employment type may play a role in the returning-to-work process. Entrepreneurs do not need to convince their employers of their work ability; thus, the decision to return to work may be mostly in their own hands. Furthermore, entrepreneurs’ return to working life may be easier in “work as you wish’-like platform-mediated occupations. Optimally, the platform economy creates new part-time employment for those with partial work abilities (e.g., [47]). However, as social security in many countries largely is based on full-time salaried employment, working part-time poses a risk of falling outside social security schemes because part-time work does not always match requirements and thresholds (e.g., having paid contributions for a certain duration within a specific period) set for accessing social security benefits [48].

On the whole, decreased work ability should not be viewed solely as an individual’s attribute or problem. Instead, it is societies’ responsibility to build working life, which includes everyone with differing capabilities. However, employers currently are not readily offering jobs to individuals whose work ability has decreased. The interviews in this study demonstrated how age discrimination, health problems and lack of up-to-date skills are common factors hindering a return to work, which may intertwine in a way so that it is not easy to distinguish them. Inclusion of individuals with health problems or disabilities is a question of both equity and provision of preventive and curative health and rehabilitation services. Supporting sustainable careers requires that workers’ skills and knowledge get updated continuously to ensure that everyone’s work ability is compatible with ever-changing demands throughout their working lives. Consequently, proactive action to support sustainable careers may decrease individuals’ feelings of being discriminated against and, thus, their intention to retire prematurely.

## 5. Conclusions

Previous research has found that individuals with decreased work ability are a heterogenous group, but this qualitative study’s main finding was that five distinctive groups could be identified among those with decreased work ability based on their orientations towards work or pension, their self-assessed health and work ability and their perceptions concerning the future: Those in the *Successful* group had managed to recover well and were motivated to return to their old jobs. Those in the *Persevering* group returned to work despite enduring pain and persistent work ability concerns. Those in the *Forward-looking* group had not recovered, nor returned to work, but were optimistically searching for new vocations in which they could flourish despite their health limitations. Those in the *Stuck* group had not recovered, nor returned to work, but unlike the previous group, they were passive and hesitant about returning to work. Those in the *Pension-oriented* group had complex problems, and they had given up hope of ever returning to work and instead clearly were inclined towards retirement.

The exit path model, constructed primarily on work ability literature, proved congruent with the different work or pension orientations found within the interviewees’ narratives. Considering that one-size-fits-all types of service models are not compatible with alleviating the wicked problem of decreased work ability, it may be useful to utilise the exit path model as a guide for designing services and communicating about them to different client groups. Those in the *Future-oriented* group should be guided towards the primary exit, which is returning to working life by finding a new suitable job that matches their decreased capabilities. However, it may be appropriate to help those in the *Pension-oriented* group exit smoothly and secure a pension without unnecessary struggles and stalemate situations related to temporary benefits. A considerable challenge lies in rehabilitating those in the *Stuck* group back into working life because their prolonged and multi-faceted challenges often require long-term collaboration among different sectors, such as health care, rehabilitation, education or training and employment services. Currently, the multi-professional service models being developed still often are fragmented, recounting the difficulty of designing and promoting cross-sectoral services.

This study indicates that in the current labour market climate, in which tough competition and age discrimination exist, it can be difficult for an individual with decreased capabilities to return to work after sickness absence. Therefore, it is important for society to bear responsibility for supporting sustainable careers, and to induce employers and employees to work together towards a more sustainable working life. It appears that a need exists for a systematic societal structure that acknowledges the capabilities of the partially disabled and steers them towards work that is adjusted to their individual needs. This is important for diminishing the current polarisation of the dichotomous working life, in which people viewed as fit for work are welcomed and those who are not 100% fit for work cannot find a place for themselves in the labour market.

## Data Availability

The data used in this study are managed by the authors. The data are not publicly available due to privacy.

## Appendix A

**Table A1:** Interviewees’ profiles.

Pseudo-nym ^1^	Age ^2^	Worker/Entrep. ^3^	Sector ^4^	Health concern ^5^	RTW ^6^	Situation ^7^	Plan ^8^	SAH ^9^ (1–5)	WA ^10^ (0–10)	Readiness ^11^(0–10)	TOIKE ^12^	Summary ^13^
Jamie	≥51	Entrep.	Agric.	Shoulder injury	Yes	Recovered, returned-to-work	Continues in old job	4	8	10	No.	Work-oriented, well recovered, motivated
River	40–50	Worker	Health	Back pain	Yes	Recovered, returned-to-work	Continues in old job	4	9	10	No.	Work-oriented, well recovered, motivated
Sam	40–50	Worker	Constr.	Back pain and burnout	Yes	Has pain, but returned-to-work	Continues in old job	3	5.5	?	Yes. No visit was booked.	Somewhat work- oriented, not fully recovered, work ability concerns
Pat	≤39	Entrep.	Constr.	Back and leg pain	Yes	Has pain, but returned-to-work	Continues in old job	2	4	?	No.	Somewhat work- oriented, not fully recovered, work ability concerns
Glen	≤39	Worker	Logistics	Shoulder injury	No	Unemployed after sick-leave	Needs a new direction	3	5.5	7.5	No, but considers.	Work-oriented, not fully recovered, hopeful
Jordan	≤39	Worker	Hosp.	Back and leg pain	No	Sick-leave	Going to re-education	4	4.5	2.5	No.	Work-oriented, not fully recovered, hopeful
Rowan	≤39	Worker	Transp.	Back pain + other	No	Sick-leave	Needs a new direction. Going to a work tryout	3	3	3.5	No, but considers.	Work-oriented, not fully recovered, hopeful
Taylor	≥51	Worker	Manuf.	Shoulder injury, dyslexia, memory disorders	No	Prolonged absence: sick-leave/ unemployed	Cannot find work	2	5	?	No.	Somewhat pension-oriented, not recovered, hesitant, passive
Blake	≥51	Worker	Manuf.	Arm injury (+ mental health)	No	Unstable work history	Temporary disability pension	3	5.5	6.5	Yes. No visit was booked.	Somewhat pension-oriented, not recovered, hesitant, passive
Frankie	40–50	Worker	Agric.	Arm & knee pain, arthritis, dyslexia	No	Prolonged absence: sick-leave/ unemployed	Cannot find work. Applying for partial disability pension	2	4	5	Yes. Has visited.	Somewhat pension-oriented, not recovered, hesitant, passive
Peyton	40–50	Entrep.	Maint.	Arm pain	No	Rehabilitee/ partial disability pension	May return to work	1	2.5	3.5	No.	Somewhat pension-oriented, not recovered, hesitant, passive
Kris	≥51	Entrep.	Constr.	Back/leg pain + other	No	Business ceased. Receives sickness allowance	Applying for disability pension	1	0	0	No.	Pension-oriented, disabled, reluctant
Mel	≥51	Worker	Manuf.	Back pain	No	Cannot return to old job due to sickness	Applying for partial disability pension	2.5	0.5	?	Yes. Has visited.	Pension-oriented, disabled, reluctant
Jo	≥51	Worker	Constr.	Knee pain	No	Attended rehabilitation. Receives sickness allowance	Applying for disability pension	2	2	0	Yes. Has a visit booked.	Pension-oriented, disabled, reluctant
Riley	≥51	Entrep.	Maint.	Back/ wrist pain + other	No	Business ceased. Receives sickness allowance	Going to rehabilitation. Applying for disability pension	1	2	0	No.	Pension-oriented, disabled, reluctant
Casey	≥51	Worker	Office	Unclear, reports to be healthy	No	Attended rehabilitation and activation measures	Cannot find work	4	9	10	No.	Pension-oriented, health status unclear, reluctant

^1^ Interviewee’s pseudonym. ^2^ Interviewee’s age group. The interviewees were categorised into three major age groups based on a categorization by Määttä [25]: early career ≤39, active career 40–50, later career ≥51. ^3^ Interviewee’s employment type, i.e., whether he/she was a worker (i.e., employee) or an entrepreneur (i.e., self-employed). ^4^ The sector in which the interviewee worked: agric. = agriculture, constr. = construction, health = health care, hosp. = hospitality, logistics = logistics, maint. = maintenance, manuf. = manufacturing, office = office work, transp. = transportation. ^5^ The disease, disorder, injury or other health concern causing decreased capacity (self-reported). ^6^ Has the interviewee returned to work (RTW)? ^7^ Current situation (recovered, has pain, returned-to-work, on a sick-leave, unemployed, prolonged absence, unstable work history, business ceased, in rehabilitation, partial disability pension, etc.). ^8^ Future labour market plan or prospects. ^9^ Self-assessed health (SAH) on a scale 1–5 (1 = very poor, 5 = very good). ^10^ Self-assessed work ability (WA) on a scale 0–10 (0 = work ability at its lifetime worst, 10 = work ability at its lifetime best). ^11^ Concerning returning to work, how ready does the interviewee feel? (0 = not at all ready, 10 = perfectly ready). ^12^ Has the interviewee been in contact with the TOIKE centre? ^13^ Summary of the interviewee’s profile, based on categorised data in this table and other answers to open-ended interview questions.
